# Circulating Tumor Cells in Right- and Left-Sided Colorectal Cancer

**DOI:** 10.3390/cancers11081042

**Published:** 2019-07-24

**Authors:** Chiara Nicolazzo, Cristina Raimondi, Angela Gradilone, Alessandra Emiliani, Ann Zeuner, Federica Francescangeli, Francesca Belardinilli, Patrizia Seminara, Flavia Loreni, Valentina Magri, Silverio Tomao, Paola Gazzaniga

**Affiliations:** 1 Department of Molecular Medicine, Circulating tumor cells Unit, Sapienza University of Rome, 00161 Rome, Italy; 2 Department of Radiological, Oncological and Pathological Sciences, Division of Medical Oncology, Sapienza University of Rome, 00161 Rome, Italy; 3 Department of Hematology, Oncology and Molecular Medicine, Istituto Superiore di Sanità, 00161 Rome, Italy; 4 Department of Surgical Sciences, Sapienza University of Rome, 00161 Rome, Italy

**Keywords:** colorectal cancer, sidedness, circulating tumor cells, epithelial-mesenchymal transition, prognosis, CellSearch^®^, ScreenCell^®^

## Abstract

Molecular alterations are not randomly distributed in colorectal cancer (CRC), but rather clustered on the basis of primary tumor location underlying the importance of colorectal cancer sidedness. We aimed to investigate whether circulating tumor cells (CTC) characterization might help clarify how different the patterns of dissemination might be relative to the behavior of left- (LCC) compared to right-sided (RCC) cancers. We retrospectively analyzed patients with metastatic CRC who had undergone standard baseline CTC evaluation before starting any first-line systemic treatment. Enumeration of CTC in left- and right-sided tumors were compared. The highest prognostic impact was exerted by CTC in left-sided primary cancer patients, even though the lowest median number of cells was detected in this subgroup of patients. CTC exhibit phenotypic heterogeneity, with a predominant mesenchymal phenotype found in CTC from distal compared to proximal primary tumors. Most CTC in RCC patients exhibited an apoptotic pattern. CTC in left-sided colon cancer patients exhibit a predominant mesenchymal phenotype. This might imply a substantial difference in the biology of proximal and distal cancers, associated with different patterns of tumor cells dissemination. The poor prognosis of right-sided CRC is not determined by the hematogenous dissemination of tumor cells, which appears to be predominantly a passive shedding of non-viable cells. Conversely, the subgroup of poor-prognosis left-sided CRC is reliably identified by the presence of mesenchymal CTC.

## 1. Introduction

There is discrepancy between the latest advances in molecular segmentation of colorectal cancer (CRC), which recently led to the consensus molecular subtyping of the disease, and the insufficient availability of biomarkers ready for routine clinical use [1]. We certainly recognize that colorectal cancer is a heterogeneous and complex disease and we recently witnessed the elegant demonstration of its spatial and temporal dynamic nature [2]. RAS gene mutations have historically been the watershed for molecular stratification of metastatic colorectal cancer (mCRC) and, so far, RAS genes mutational analysis is the only discriminant between frontline treatment options in this setting. In the last few years, we have made progress in the search for strategies to repeatedly monitor the molecular makeup of colorectal cancer along its Darwinian temporal evolution and we gained preliminary, although promising, elements on the possibility to adapt therapies on the basis of the continuous mutability of colorectal cancer clones [3]. Evidence is emerging that molecular alterations are not randomly distributed in colorectal cancer, but rather clustered on the basis of primary tumor location and underlie the prognostic and predictive significance of colorectal cancer sidedness. Colorectal cancers indeed exhibit differences in epidemiology, clinical presentation, and outcomes depending on the location of the primary tumor. It has been reported that right-sided tumors have a lower incidence, a more advanced stage at presentation, and are associated with worse prognosis compared to left-sided colorectal tumors [4]. Further than RAS mutational status and EGFR activation, biological reasons behind the differences between left- and right-sided colorectal cancers are laid aside. Several publications have demonstrated that distinct metastatic patterns in colorectal cancer patients exist based on primary tumor location [5,6]. This might be attributable to a number of reasons, including the embryological origin of proximal and distal cancers, the anatomical location and venous drainage, and the biology of right-sided and left-sided cancers. Prominent differences have been also demonstrated in terms of tumor microenvironment, which results to be inflamed and with marked stromal infiltration in left cancers as compared to right cancers, which in turn are characterized by lower inflammatory status and higher expression of pro-angiogenic factors. This might also explain the different pattern of response to biological agents, with left-sided colorectal cancer (LCC) more prone to respond to anti-EGFR therapies and right-sided colorectal cancer (RCC) more responsive to antiangiogenic drugs. Cancer-specific molecular alterations and tumor microenvironment structure might both affect the dissemination pattern of cancer cells and ultimately determine the outcome of patients. The dissemination of cancer cells from primary tumors into distant sites represents the first event in the multistep process known as the invasion–metastasis cascade. Individual cancer cells and multi-cellular cohorts (clusters) arising from primary tumors intravasate and travel to distant tissues thus representing an intermediate between primary tumors and eventually formed metastatic colonies [7]. We have previously demonstrated that the presence of even a single circulating tumor cell in the peripheral blood of patients with mCRC significantly affects their prognosis [8]. Of additional relevance is the well-proven heterogeneity in the biological properties of either single or clustered CTC, which often exhibit various combinations of epithelial and mesenchymal traits [9]. Hence, a more in depth analysis of the biological programs activated in CTC might help clarify how different the patterns of dissemination might be relative to the behavior of primary tumors. The primary aim of the present study was to evaluate whether the presence of circulating tumor cells, as a surrogate of tumor in the bloodstream, might dichotomize according to sidedness and whether this might have a prognostic impact. The secondary aim was to investigate whether CTC isolated from proximal and distal colorectal cancers might differ in their biological features, specifically referring to epithelial-mesenchymal transition (EMT)-related phenotype. 

## 2. Results

### 2.1. Enumeration of CTC According to Tumor Sidedness

Eighty-four metastatic colorectal cancer patients were included in this retrospective analysis. The whole population was divided into three subgroups according to primary tumor sidedness: 24 right-sided CRC, 31 left-sided CRC, and 29 rectal cancers. The demographic and clinicopathological characteristics of the whole population are shown in Table 1. 

We report here that CTC were not uniformly distributed in the three subgroups of patients and were found in 46%, 39%, and 38% of patients with primary RCC, LCC, and rectal cancer, respectively. This difference was not found to be statistically significant (*p* = 0.8). Some degree of heterogeneity was also observed in terms of number of CTC. Indeed, the highest median number of CTC was observed in the group of patients with RCC (6.75, range 0–67), as compared to LCC (1.29, range 0–9), and rectal cancers (2.68, range 0–37). While the comparison between the three groups was found not statistically significant by ANOVA test (*p* = 0.12), the comparison between paired groups demonstrated a difference in CTC number distribution only between RCC and LCC. This difference was found to be statistically significant (*p* = 0.03).

**Figure 1:**
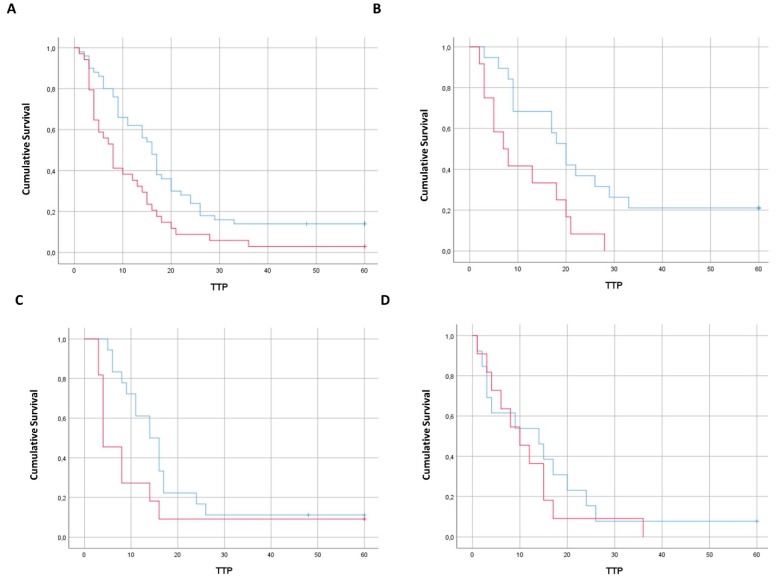
Prognostic impact of CTC on time to progression (TTP). Panel (**A**): Comparison in TTP between CTC-positive vs. CTC-negative samples (all mCRC pts). Panel (**B**): Comparison in TTP between CTC-positive vs. CTC-negative samples in left colon cancer pts (*p* = 0.009). Panel (**C**): Comparison in TTP between CTC-positive vs. CTC-negative samples in rectal cancer pts (*p* = 0.058). Panel (**D**): Comparison in TTP between CTC-positive vs. CTC-negative samples in right colon cancer pts (*p* = 0.5).

Unexpectedly, the highest prognostic impact was exerted by CTC in left-sided primary cancer patients, even though the lowest median number of cells was detected in this subgroup of patients. Indeed, the prognostic impact of CTC in terms of time to progression (TTP) reached a robust statistical significance only in the subgroup of patients with primary left colon cancer (11.1 months in CTC positive vs. 25.6 months in CTC negative patients, *p* = 0.009), while being on the threshold of significance in rectal cancer patients (11.6 months in CTC positive vs. 18 months in CTC negative patients, *p* = 0.058) and not wholly significant in patients with primary right colon cancer (11.5 months in CTC positive vs. 15.2 months in CTC negative patients, *p* = 0.5) (Figure 1). 

### 2.2. Apoptotic Morphology of CTC 

**Figure 2:**
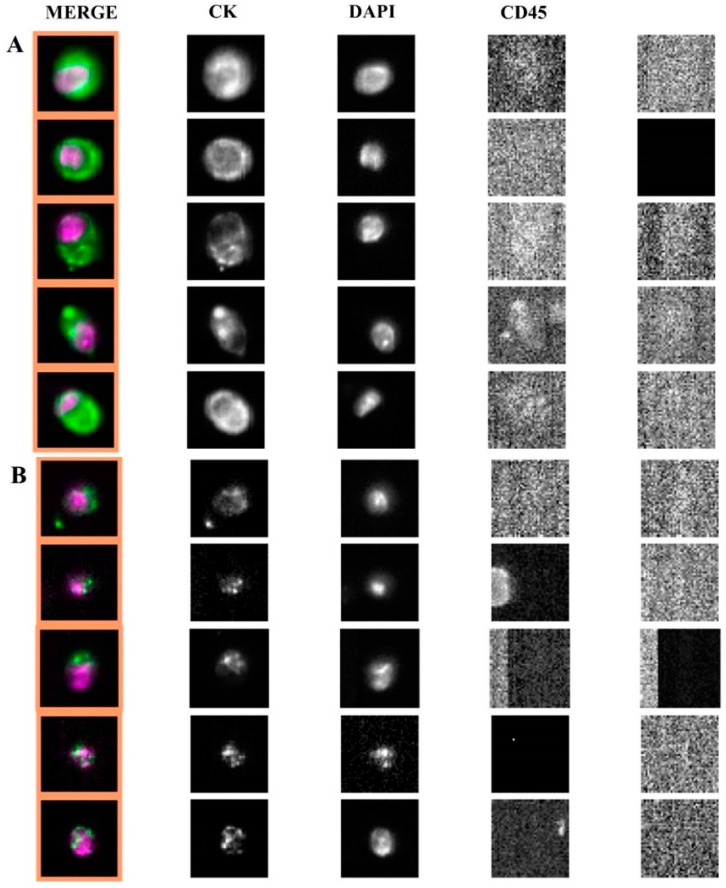
Images of intact CTC (panel **A**) and apoptotic CTC (panel **B**) isolated through CellSearch^®^ from left-sided and right-sided colorectal cancers, respectively.

In the whole population of 84 patients, we further retrospectively review all the archived images of the CellSearch^®^ analyses in order to investigate whether an apoptotic morphological pattern could be differentially observed in CTC from RCC patients as compared to LCC and rectal cancer patients. Apoptotic CTC were defined as all EpCAM+, CK+, DAPI +, CD45- CellSearch^®^ events with altered morphological parameters such as speckled pattern of keratin staining and/or fragmented or disintegrated nuclei [10]. We found that 130/162 CTC (80%) visualized in RCC patients exhibited a clear apoptotic morphological pattern differently from left-sided colon cancers and rectal cancers, where only 12/118 (10%) displayed apoptotic CTC (Figure 2). 

### 2.3. Epithelial-Like and Mesenchymal-Like Features of CTC

**Figure 3:**
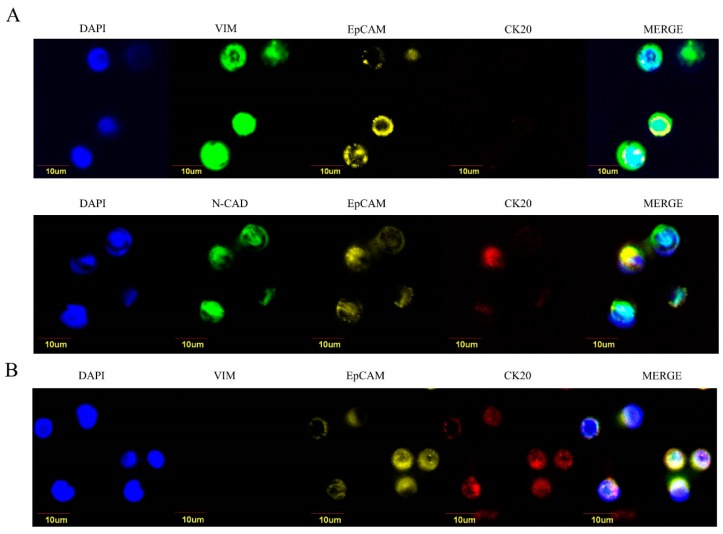
Epithelial-like and mesenchymal-like CTC isolated from left-sided (panel **A**) and right-sided (panel **B**) colorectal cancers. Panel **A**: Epithelial-like CTC analyzed by immunofluorescence for EpCAM (yellow), cytokeratin 20 (CK20, red), and vimentin/N-cadherin (VIM/N-CAD, green). In blue, nuclei are visualized. Original magnification 60×. Panel **B**. mesenchymal-like CTC analyzed by immunofluorescence for EpCAM (yellow), cytokeratin 20 (CK20, red), and vimentin (VIM, green). In blue, nuclei are visualized.

In order to provide a possible explanation for the unpredictable strongest prognostic significance of CTC in LCC patients, we performed a second analysis to gather data on the biology of CTC. To this purpose, we analyzed 24 patients (15 retrospectively and 9 prospectively enrolled) in order to perform the molecular characterization of CTC isolated from whole blood through a filtration device (ScreenCell^®^ Cyto), which allows the separation of live cells for downstream cytology studies. For each filter, three microscopic fields were analyzed. Seven RCC, nine LCC, and eight rectal cancers were analyzed for epithelial-like and EMT-like markers. Vimentin and N-cadherin were chosen as mesenchymal-like markers, and CK20 was selected as colon cancer-specific epithelial marker. Despite the limited number of samples available, we found that CTC isolated from RCC patients exhibited a predominant epithelial-like phenotype (EpCAM+, CK20+, vimentin−/N-cadherin−) as compared to the mesenchymal-like traits observed in CTC from LCC cancer patients (EpCAM+/−, CK20−, vimentin+/ N-cadherin+) (Figure 3). 

The percentage of epithelial-like and mesenchymal-like CTC in RCC, LCC, and rectal cancers are illustrated in Table 2. 

## 3. Discussion

This is the first publication reporting on the detection rate and the prognostic significance of CTC according to primary tumor sidedness in metastatic colorectal cancer patients. Although the term “colorectal cancer” is currently referred to a single tumor type, increasing evidence is emerging concerning the prognostic impact of primary tumor sidedness. A recent meta-analysis of 66 clinical studies compared the overall survival of RCC versus LCC in over 1.4 million patients and demonstrated a 20% reduced risk of death for patients whose tumors arise from the left side [11]. To date, the molecular background of proximal and distal colorectal cancers has been only preliminary unraveled and translational efforts to gain knowledge about the biological underpinnings of colorectal cancer sidedness are critically important. It has been shown that baseline detection of CTC in metastatic colorectal cancer is an independent prognostic factor for progression-free survival (PFS) and overall survival (OS) with CellSearch^®^ [12]. We confirmed here that the presence of CTC is a significant prognostic factor in metastatic colorectal cancer patients regardless of tumor sidedness. The whole population was then sub-grouped according to primary tumor sidedness. In particular, left-sided colon cancers and rectal cancers were considered separately in this retrospective evaluation, in line with recently released data supporting the colorectal cancer “three entities” hypothesis [13,14,15]. In our series of patients, CTC were not uniformly distributed in the three subgroups, showing the highest prognostic impact in patients with left-sided colon cancer, while being the lowest in number in this subgroup of patients. We hypothesized that this unanticipated result could reflect a difference at the molecular level between CTC shed from left-sided as compared to right-sided primary tumors. Indeed, we found that CTC exhibit phenotypic heterogeneity, with a predominant mesenchymal phenotype found in CTCs from distal compared to proximal primary tumors. It is well recognized that the EMT program enables epithelial cancer cells to acquire properties that are critical to invasion and metastatic dissemination, such as increased motility, invasiveness, and the ability to degrade components of the extracellular matrix. The EMT program that allows cancer cells to disseminate from a primary tumor also promotes their self-renewal capability, usually depicted as the defining trait of cancer stem cells. Such EMT process seems almost invariably triggered by heterotypic signals, including TGF-β, Wnt, and interleukins, which cancer cells receive from the tumor reactive stroma, which thus plays a substantial role in dictating cancer progression [16]. Tumor cells can reach the circulation by either active invasion, which requires the acquisition of certain mesenchymal traits, or by passive shedding, due to cancer cell pushing by tumor expansive growth and facilitated by the abundance of highly abnormal blood vessels. Tumors are likely able to use both active and passive methods to enter the vasculature, depending on the site of tumor initiation, the aggressiveness of tumor cells, and the tumor micro-environmental conditions. It has been reported that most of the cancer cells that entered into the vasculature by passive shedding are non-viable cells, thus incapable of completing the efficient colonization of distant sites [17]. Distinct metastatic patterns in colorectal cancer patients based on primary tumor location have been recently demonstrated, with higher rates of liver and lung metastases in left-sided colon cancers and rectal cancers, respectively, as compared to right-sided tumors, which appear to be associated with higher rates of peritoneal metastases. We could hypothesize that proximal and distal colorectal cancers may also differ in the early steps of the metastatic cascade and that alternative modalities of tumor cells intravasation might be adopted, depending on primary tumor location. As far as we know, substantial differences exist in the tumor microenvironment of distal and proximal colorectal cancer, with the former being inflamed and with marked stromal infiltration and the latter characterized by lower inflammatory status and higher expression of pro-angiogenic factors [18]. With this in mind, we could envisage that cancer cells from primary distal cancers receive abundant signals from the surrounding reactive stroma, which are able to activate their latent EMT programs and equip them with the ability to actively intravasate into the circulation. Conversely, proximal cancers, which are frequently larger in size and plenty of disorganized and leaky blood vessels, might release into the circulation a higher number of cancer cells, which are not necessarily viable and able to sustain the following steps of the metastatic cascade. As expected, we found that the vast majority of CTC in RCC patients exhibited a clear apoptotic pattern, thus, possibly providing the rationale for the limited prognostic impact of these cells in patients with primary proximal cancers. Although we did not use any apoptosis-specific marker to identify apoptotic CTC, several reports have described apoptotic CTC as a specific CTC subtype well identifiable at CellSearch^®^ [10,19]; they are characterized by altered morphological parameters such as speckled pattern of keratin staining and/or fragmented or disintegrated nuclei.

Our data suggest that the poor prognosis of right-sided colorectal cancer might not be determined by the hematogenous dissemination of tumor cells, which appears to predominantly be a passive shedding of non-viable cells in the blood vessels. We also demonstrate that a subgroup of poor-prognosis left-sided colon cancer exists, which is reliably identified by the presence of CTC in the blood vessels. Particularly, CTC found in left-sided colon cancer patients exhibit a phenotype with different levels of mesenchymal differentiation. This might imply a substantial difference in the biology of proximal and distal cancers, mainly related to the tumor microenvironment and strongly associated with different patterns of tumor cells dissemination from primary tumors. Although EMT is certainly triggered by stromal signals, EMT-specific traits in CTC, which are someway "stromal independent", might indicate that cancer cells in the blood are able to transcriptionally control their nature by cell-autonomous mechanisms, as recently advocated by the new CRIS-B subtype of colorectal cancer [20]. The main limitation of the study is the small population of samples available for molecular analysis, and our results need to be confirmed in a larger cohort. Investigating the intracellular properties of CTC, in their fluid microenvironment, might contribute to define how and to what extent cancer cell-specific traits contribute to the creation of accurate molecular subtypes of CRC and to the definition of reliable prognostic indicators.

## 4. Materials and Methods 

### 4.1. CTC Enumeration 

We retrospectively analyzed patients with mCRC who had undergone standard baseline CTC evaluation through the CellSearch^®^ platform (Menarini Silicon Biosystems, Castel Maggiore, Bo, Italy) before starting any first-line systemic treatment between 2010 and 2015. Informed consent had been obtained in all patients. A total of 84 mCRC patients were included in this retrospective study. Information on primary tumor location was obtained from the original pathology reports. Primary tumors located in the proximal two-thirds of the transverse colon, ascending colon, and caecum were coded as right-sided. Tumors located in the distal third of the transverse colon, splenic flexure, descending colon, and sigmoid colon were categorized as left-sided. The protocol had been approved by Ethical Committee of Policlinico Umberto I (protocol n. 668/09, 9 July 2009; amended protocol 179/16, 1 March 2016). 

From each patient, 7.5 mL of peripheral blood was collected in CellSave preservative tube (Menarini Silicon Biosystems) containing EDTA and a cell fixative, maintained at room temperature and processed within 72 h. The CTC enumeration was carried out through the CellSearch^®^ system, employing CellSearch^®^ Epithelial Cell Kit, which contains a ferrofluid-based capture reagent and immunofluorescent staining reagents. Briefly, CTC were first enriched from 7.5 mL of whole blood by anti-EpCAM-antibody-coated ferrofluid reagent and subsequently stained for cytokeratins (CK), 4’-6-Diamidino-2-phenylindole (DAPI), and CD45. An event was classified as CTC when exhibiting the phenotype EpCAM+, CK+, DAPI+, and CD45-. Apoptotic CTC were defined as all EpCAM+/CK+/CD45-cells characterized by altered morphological parameters such as speckled pattern of keratin staining and/or fragmented or disintegrated nuclei [10].

### 4.2. Epithelial-Like and Mesenchymal-Like CTC

To isolate CTC for cytological studies, ScreenCell^®^ Cyto kit (ScreenCell, Sarcelles, France) was used, an EpCAM-independent device allowing size-based separation of CTC from whole blood. A total of 24 patients were analyzed. In order to fix the cells and to lyse red blood cells (RBC), 3 mL of blood was diluted in 4 mL of filtration buffer (FC). After 8 minutes of incubation at room temperature, 7 mL of diluted sample was added into device tank and filtered under a pressure gradient using a vacutainer tube. Filtration was usually completed within 3 minutes. After washing with PBS to remove RBC debris, each filter was left on absorbing paper to dry at room temperature. After hydration with Tris-buffered saline (TBS) for 10 minutes, the filters were incubated in a humid chamber overnight at 4 °C with the following primary antibodies: Goat polyclonal anti-cytokeratin (CK) 20 (N-13, 1:100; Santa Cruz Biotechnology Inc., Rockford, IL, USA), mouse monoclonal anti-epithelial cell adhesion molecule (EpCAM) (VU1D9, 1:100; Invitrogen, Rockford, IL, USA), and rabbit polyclonal anti-vimentin (H-84, 1:100; Santa Cruz Biotechnology Inc.) or rabbit polyclonal anti-N-Cadherin (D4R1H, 1:100; Cell Signaling Technology). The next day, filters were washed twice in PBS and then incubated with donkey anti-goat Alexa Fluor 647 (Molecular Probes, Eugene, OR, USA) secondary antibody for 45 minutes at room temperature in the dark. After washing in PBS, filters were incubated with goat serum (1:10) for 30 minutes to reduce cross reactivity between goat anti-rabbit, goat anti-mouse, and donkey anti-goat secondary antibodies. Serum was removed without washing and filters were incubated with goat anti-rabbit Alexa Fluor 488 and goat anti-mouse Alexa Fluor 555 (Molecular Probes) for 45 minutes at room temperature in the dark. Nuclei were stained with 4’,6-diamidino-2-phenylindole (DAPI; Invitrogen) for 15 minutes at room temperature. All antibodies were dissolved in PBS containing 3% bovine serum albumin (BSA), 3% fetal bovine serum (FBS), 0.001% NaN3 and 0.1% Triton X-100. Finally, the filters were mounted with Prolong-Gold Antifade (Invitrogen) on slides and analyzed using a FV1000 Confocal microscope (Olympus FV1000) equipped with a 60× oil immersion objective. Markers levels were evaluated based on immunofluorescence staining intensity. Results were provided as a discrete nominal (positive/negative) score. 

Chi Square test was used to assess the difference of CTC positive cases according to tumor sidedness. The one-way analysis of variance (ANOVA) and T student’s test were used to assess the statistical significance of CTC number distribution in RCC, LCC, and rectal cancer. Survival analysis was conducted with the Kaplan–Meier method, yielding median survival times (95% confidence intervals) and comparing survival curves with the log-rank test. Statistical significance was set at the 2-tailed 0.05 level. Computations were performed with IBM SPSS Statistics 24.0. 

## 5. Conclusions

Circulating tumor cells from distal compared to proximal colorectal tumors display quantitative and qualitative heterogeneity, with rare cells and predominant mesenchymal phenotype found in CTC isolated from left-sided tumors. Right-sided tumors are characterized by a high percentage of apoptotic CTC. The EMT-like features of CTC in left-sided colon cancer might account for the poor prognosis observed in the subgroup of CTC positive patients.

## Figures and Tables

**Table 1:** Demographic and clinicopathological characteristics of patients analyzed for circulating tumor cells (CTC) enumeration (CellSearch^®^).

Characteristics	No. of Patients (*n* = 84)
Sex	
Male	50
Female	34
Primary tumor location	
Right	24
Left	31
Rectum	29
Stage of disease	
Metastatic	84
KRAS status (tumor tissue)	
Wild type	31
Mutant	29
Unknown	24

**Table 2:** Percentage of epithelial-like and mesenchymal-like CTC in right-sided cancers (RCC), left-sided cancers (LCC), and rectal cancers.

Patient n.	Tumor Location	n. CTC (ScreenCell)	Epithelial-Like CTC (%)	Mesenchymal-Like CTC (%)
1	right	20	16 (80)	4 (20)
2	right	5	5 (100)	0 (0)
3	right	16	10(63)	6 (37)
4	right	25	20 (80)	5(20)
5	right	8	7 (87)	1 (13)
6	right	30	25 (83)	5 (17)
7	right	27	22 (81)	5 (19)
8	left	4	0 (0)	4 (100)
9	left	4	1 (25)	3 (75)
10	left	10	1 (10)	9 (90)
11	left	11	4(36)	7 (64)
12	left	6	2(33)	4 (67)
13	left	16	5(31)	11 (69)
14	left	7	0(0)	7 (100)
15	left	8	2(25)	6 (75)
16	left	10	2(0)	8 (100)
17	rectum	5	0(0)	5 (100)
18	rectum	10	2(20)	8 (80)
19	rectum	14	2(14)	12 (86)
20	rectum	7	2(29)	5 (71)
21	rectum	10	0 (0)	10 (100)
22	rectum	6	1 (17)	5 (83)
23	rectum	8	0 (0)	8 (100)
24	rectum	3	0 (0)	3 (100)
